# Different reprogramming propensities in plants and mammals: Are small variations in the core network wirings responsible?

**DOI:** 10.1371/journal.pone.0175251

**Published:** 2017-04-06

**Authors:** Victor Olariu, Julia Nilsson, Henrik Jönsson, Carsten Peterson

**Affiliations:** 1 Computational Biology and Biological Physics, Lund University, Lund, Sweden; 2 Center for Models of Life, Niels Bohr Institute, University of Copenhagen, Copenhagen, Denmark; 3 Sainsbury Laboratory, University of Cambridge, Cambridge, United Kingdom; 4 Department of Applied Mathematics and Theoretical Physics (DAMTP), University of Cambridge, Cambridge, United Kingdom; University of the Basque Country, SPAIN

## Abstract

Although the plant and animal kingdoms were separated more than 1,6 billion years ago, multicellular development is for both guided by similar transcriptional, epigenetic and posttranscriptional machinery. One may ask to what extent there are similarities and differences in the gene regulation circuits and their dynamics when it comes to important processes like stem cell regulation. The key players in mouse embryonic stem cells governing pluripotency versus differentiation are Oct4, Sox2 and Nanog. Correspondingly, the WUSCHEL and CLAVATA3 genes represent a core in the Shoot Apical Meristem regulation for plants. In addition, both systems have designated genes that turn on differentiation. There is very little molecular homology between mammals and plants for these core regulators. Here, we focus on functional homologies by performing a comparison between the circuitry connecting these players in plants and animals and find striking similarities, suggesting that comparable regulatory logics have been evolved for stem cell regulation in both kingdoms. From *in silico* simulations we find similar differentiation dynamics. Further when in the differentiated state, the cells are capable of regaining the stem cell state. We find that the propensity for this is higher for plants as compared to mammalians. Our investigation suggests that, despite similarity in core regulatory networks, the dynamics of these can contribute to plant cells being more plastic than mammalian cells, *i*.*e*. capable to reorganize from single differentiated cells to whole plants—reprogramming. The presence of an incoherent feed-forward loop in the mammalian core circuitry could be the origin of the different reprogramming behaviour.

## Introduction

The differences in reprogramming competence between plants and animals might not be surprising, as the survival of plants requires more plasticity regarding shape and form given their fixed location. However, the underlying mechanisms for these differences are still obscure. Understanding the origin of these differences would be very elucidating when comparing the different kingdoms and could be of paramount value for further development of efficient reprogramming recipes in mammalian systems with impact on tissue regeneration in health care. The origin of these differences between the kingdoms could be of transcriptional, epigenetic or signalling nature or combinations thereof. In here we focus on transcriptional regulation as the available epigenetic and signalling data do not provide a clear answer to our question.

To this end we will consider the *in silico* dynamical properties of the core transcriptional architectures for Oct4, Sox2, Nanog [1–3] inside embryonic stem cells (ESC) and WUSCHEL (WUS), CLAVATA3 (CLV3) for shoot apical meristem (SAM) respectively [4–6]. We put forward computational models for ESC and SAM exhibiting bistability. The two stable states are linked to the differentiated and pluripotent cell state respectively. We envisage reprogramming and differentiation as dynamical transitions between the two stable states, and analyse the differences in the transition dynamics between the mammalian and plant networks.

Prior to presenting the dynamical models and their results, we briefly review what is known from epigenetics and cell signalling in the context of plant and mammalian stem cell differentiation and reprogramming.

Chromatin modifications have been identified as important for differentiation and reprogramming. Chromatin remodelling is key to reprogramming in mammalian stem cell systems [7, 8]. In the core Oct4/Sox2/Nanog architecture for embryonic stem cells (ESC) Oct4 opens up the Nanog region by turning on the Jmjd1a and Jmjd2c demethylases [9]. The H3K9me3 mark is responsible for making the chromatin of most pluripotency genes inaccessible for binding. The demethylases activated by Oct4 lead to global H3K9me3 depletion improving cell reprogramming [10, 11]. Nanog is important for the ESC state as it facilitates transition of partially reprogrammed cells towards ground state [12]. Moreover, Nanog enhances the expression of Oct4 by recruiting Tet1, which modulates DNA methylation levels at CpG- rich promoters, promotes transcription of pluripotency factors and participates in the repression of Polycomb-targeted developmental regulators [13, 14].

In the Shoot Apical Meristem (SAM), stem cells are maintained throughout the life of the plant. Cells are stuck together via cell walls and stem cells situated at the very apex are pushed, via growth, to the periphery of the apex where differentiation starts. At the core of the regulation of stem cell maintenance is the homeodomain transcription factor WUSCHEL (WUS) sufficient and necessary for stem cell activity [15]. WUS is expressed in the central part of the meristem, can move between cells [16, 17], and has been shown to activate stem cell genes and repress (potentially with co-factors) differentiation genes [18]. WUS activates CLAVATA3 (CLV3) in the stem cells, which is part of negatively regulating WUS. Together, this forms a negative feedback regulation assumed to be at the core of the stem cell maintenance regulation. In flower primordia there is a transient stem cell activity regulated by the same CLV3/WUS network as in the SAM before differentiation into specialized flower organs [4]. The inactivation of WUS expression here results from an interplay between transcriptional and epigenetic regulation [19, 20]. While factors with chromatin remodelling effects within the SAM have so far been identified [21, 22], no specific mechanistic relationships with the core network as for the mammals have so far been mapped out. This asymmetry in detailed knowledge of epigenetic regulation between the two kingdoms could be due to the surge in focus when it comes to reprogramming with regenerative medicine as a goal.

In the context of stem cell reprogramming, also external signals have been proposed to be important [23–26]. Plant cells react to relative levels of the plant hormones auxin and cytokinin for regeneration of shoot or root tissues from cells extracted from different organs [23]. The plant protocol does not require manipulations in terms of external over-expression of certain genes in the differentiated cell in contrast to the mammalian case [24]. In the SAM, cytokinin has been suggested to directly impact the CLV3/WUS system to activate the central stem cell core [27], while high auxin levels are important for the initiation of new organs at the periphery [28]. Also microRNAs have been proposed as a main intercellular signalling molecule for several developmental processes in plants, including the regulation of stem cell maintenance [25]. In short, plant stem cell regulation has been evolved to heavily make use of intercellular communication within its niche. So far, transcription factor transfer between cells has not been identified in mammalian cells, though it has been shown that Oct4 forms a complex with E-cadherin and β-catenin at the membrane of the mouse ESC playing a central role in pluripotency [26]. In conclusion, plants’ needs for responding to the environment, partly connected to signalling molecules and lack of cell migration, can be connected to a more abundant use of cell-to-cell communication compared to animals. Whether this is due to a more complex extracellular matrix in the latter or this is enough to explain differences in plasticity remains to be understood.

## Results

### Direct comparison between the core regulatory networks in plants and mammals identifies similarities

#### The shoot apical meristem model

**Fig 1:**
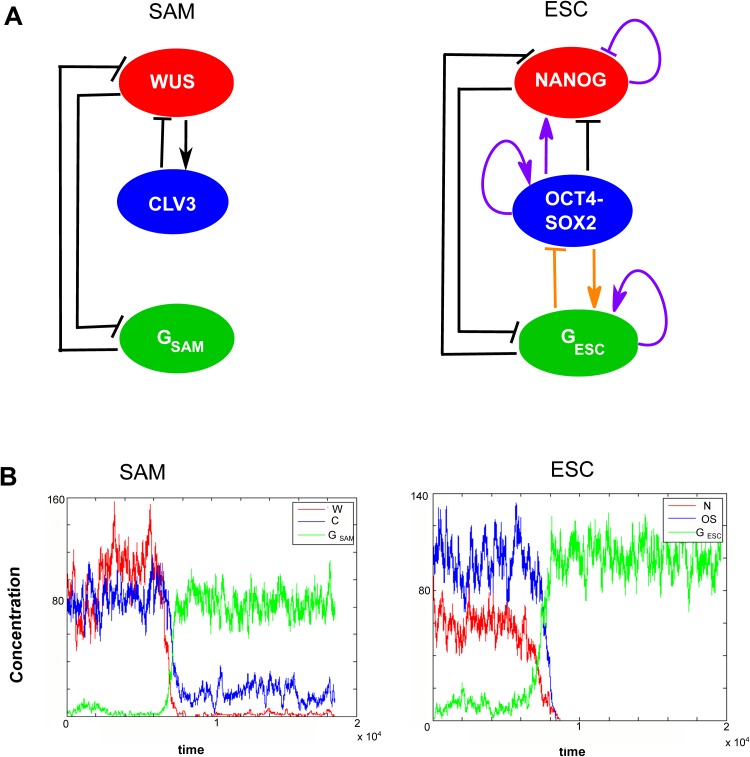
Comparison between network topology and differentiation dynamics of the two single cell minimal models of SAM [18] and ESC [38]. A) Gene regulatory networks where black interactions are common to both models, purple interactions are specific for ESC and the orange interactions indirectly exist in the plant stem cell (SAM) dynamics. For model and parameters descriptions see Methods section. B) Examples of time series results of differentiation simulation with SAM and ESC model.

We define a model using the main components of the SAM dynamics (Fig 1A). For the stem cell activity these are three CLAVATA genes (CLV1, 2 and 3) [6, 29, 30], and WUSCHEL [31]. CLV3 is localized in the central zone [27] and is recognized as a marker of stem cell identity whereas WUS is expressed below in the organizing centre [13]. The three CLV genes have been suggested to function in the same pathway to control meristem development [5, 29, 30] by repressing WUS [32, 33]. WUS, on the other hand, induces the expression of CLV3 [33] and thereby stem cell identity.

G_SAM_ represents the genes expressed in the peripheral zone in the differentiated cells. In this study, the interactions for G_SAM_ are based on KAN1, since perturbation data are available for this gene [18]. However several genes behave in a similar manner, some of which were analysed for WUS changes in [18]. The network topology built based on these experimental observations is shown in Fig 1A. It should be noted that in here we exclude all transports between expression domains in the SAM model and focus on the intracellular expression states.

#### The embryonic stem cell model

In the embryonic stem cell the core of the gene regulatory network consists of the genes OCT4, SOX2 and NANOG [1–3]. These three transcription factors maintain the pluripotent state. The regulation of the three main genes by the transcription factors OCT4 and SOX2 is attained through the formation of the two into a heterodimer—an OCT4/SOX2 complex [34]. OCT4/SOX2 induces its own transcription [34] whereas NANOG, according to recent findings, represses its own expression [35, 36]. In [9] an epigenetic effect is suggested in which OCT4 activates a component that opens up, among other genes, NANOG. This means that the regulatory region of NANOG is inaccessible in the absence of OCT4. Other than the three genes mentioned above the OCT4/SOX2 complex also induces FGF4, which promotes differentiation through NANOG repression [37]. This model is presented in [38] and it also includes differentiation gene G. Possible candidates for gene G are Sox17 and Gata6.

In this study, we propose a simplified ESC network compared to the one in [38] where we remove FGF4 and include instead direct repression of NANOG by OCT4/SOX2 complex (Fig 1A).

Assuming that OCT4/SOX2 translates into CLV3, NANOG into WUS and G_ESC_ into G_SAM_ a comparison between the models reveals that three interactions, marked in black in Fig 1A, are common to both models. All self-regulatory interactions and induction of NANOG by OCT4/SOX2 are specific to the ESC model (purple in Fig 1A) and the interactions between G_ESC_ and OCT4/SOX2 are indirect reactions in the SAM model (orange in Fig 1A). The repression of OCT4/SOX2 by G_ESC_ exists in the SAM network through G_SAM_ represses WUS, which activates CLV3, while CLV3 activates G_SAM_ by repressing WUS, which is a G_SAM_ repressor. Similarly, the direct activation of the CLV3 from WUS in the plant network is included in the ESC system by NANOG repressing the repressor of OCT4-SOX2. In summary, we find striking similarities between SAM and ESC model with regard to majority of the regulatory interactions if indirect interactions are accounted for although the ESC system has a few additional identified regulatory interactions.

### Dynamical properties of transcriptional stem cell regulation suggest differences between plants and mammals in transition propensities from differentiated to stem cell states

The similarities in molecular mechanisms identified and the structure of the transcriptional regulatory modules cannot explain the differences in reprogramming competence between plants and mammals. Hence it is tempting to pursue dynamical modelling in order to identify such differences due to the complexity resulting from the differences of interactions (Fig 1A).

Using simplified stochastic computational models for the mammalian [38] and plant stem cell [18] dynamics on the single cell level reveal great similarity in the dynamical behaviour of the systems (Methods, Fig 1B). The single cell approach is a simplification, as intercellular signalling within the stem cell niches are replaced by externally applied signals, but still captures the main dynamics and attractors of the systems [18, 38]. In both the animal and plant cases, the systems have natural differentiation dynamics where they spontaneously jump between the stem cell state and the differentiated state, while they less frequently (typically never in our long simulations) spontaneously jump from the differentiated state to the stem cell state (Fig 1B). This holds true for a range of parameter values selected for additional independent perturbation dynamics (Methods, [18, 38]). The similarity may reflect the core mutual inhibition between stem cell factors and differentiation factors described above that has been identified in both animals and plants [18, 38].

**Fig 2:**
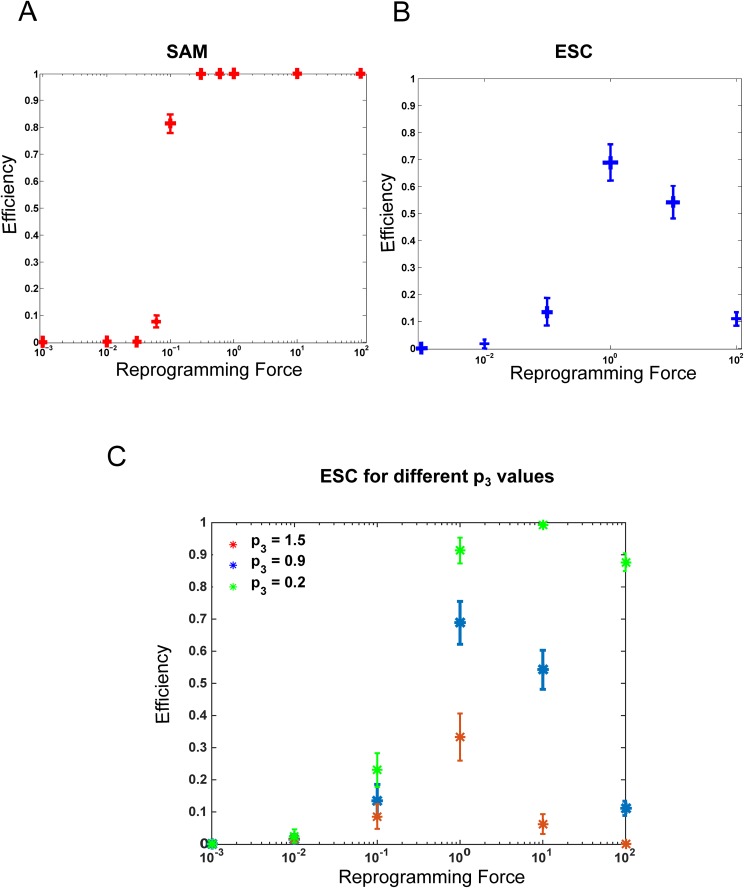
Reprogramming efficiency by over-expressing Oct4 and WUS respectively. Over-expression was implemented by adding a constant production (reprogramming force) to the equation for OCT4/SOX2 mRNA rate of change in the ESC model and correspondingly for WUS in the SAM model. If the addition of this constant resulted in a switch of the system to a pluripotent state we considered the reprogramming to be successful. The results are based upon monitoring the reprogramming success in 100 independent stochastic Gillespie runs. A) Reprogramming efficiency for different levels of WUS overexpression in the plant SAM model. (B) Reprogramming efficiency for the mammalian ESC model for different levels of Oct4 overexpression. Note the ease by which the plant-differentiated cell is reprogrammed as compared to the mammalian cell, since the latter requires the reprogramming force to be within a certain interval. (C) Reprogramming efficiency in the ESC model for different values of OCT4 overexpression when incoherence parameter (p_3_) takes three different values.

Given this similarity it is of interest to also investigate how the circuits behave when simulating reprogramming experiments, within the parameter ranges of the models. The ESC and SAM models give rise to very different reprogramming behaviour, Fig 2. The SAM model efficiency has a sharp transition from zero to one, where one means that all simulations led to successful reprogramming. The ESC model, on the other hand, has a reprogramming efficiency that peaks for some reprogramming forces and then decreases. This corresponds well to the experimental and computational result that reprogramming works best for a limited range of over-expression [38, 39, 40]. In plants, ectopic WUS, either directly or via removing the function of CLV3 has been used to create ectopic stem cells in vivo [16, 41, 42]. In the ESC system over-expressing Oct4 would represent reprogramming experiments in the simplified model [43, 44, 45]. Strikingly, the plant system reaches its maximum in de-differentiation earlier as compared to the mammalian system (Fig 2). More importantly, the drop in the latter for high over-expression is consistent with experimental results [38, 39] and is a consequence of the attractor structure of the non-linear dynamics for this model (Fig 2B). While this might indicate a failure of de-differentiation at high levels or a push of the system into another differentiated state, it clearly predicts that a more specific treatment is needed for de-differentiation in the mammalian system [18, 38].

In summary, our simplified regulatory network models indicate that although they have similar interactions and differentiation dynamics, the models captures the increased difficulty of reprogramming in the ESC cells compared to plant stem cells.

### Adjusting the interaction strengths in the ESC network can increase the propensity for reprogramming

Furthermore, we attempted to elucidate which couplings in the ESC network that might be responsible for the difference in reprogramming efficiency between the SAM and ESC systems. To this end we performed simulations where we modified one parameter at a time while keeping the others unchanged and assessed the impact on reprogramming efficiency

The parameters that influenced the most the reprogramming outcomes are p_1_ and p_3_ corresponding to the incoherent feed-forward motif for Oct4 regulating Nanog (Table C in S1 File). If p_1_ is reduced, corresponding to the activating interaction, Nanog cannot be induced by Oct4 and reprogramming is lost. The parameter p_3_ is representing the Nanog repression by Oct4. Fig 2C shows reprogramming efficiency for various levels of the reprogramming force when Oct4 represses Nanog with three different strength values. The results show that reducing Nanog repression by Oct4 leads to an ESC reprogramming efficiency similar to that of SAM (Fig 2C green data). Of note is that Oct4 repression of Nanog can be indirect through *e*.*g*. Fgf4. However in this study we considered a simplified network topology and assume that such interactions are captured in the Oct4 directly repressing Nanog. In plants, the corresponding reduction of the repression from CLV3 on WUS leads to increased number of stem cells. In particular, induced silencing shows spontaneous reprogramming of meristematic cells [46].

In summary, the need of specific treatment in the mammalian system might be due to the presence of incoherent loops between pluripotency factors. Our simulations show that the removal of incoherence in the ESC system drastically reduces the differences in reprogramming efficiencies in the two kingdoms.

### Reprogramming time differences

**Fig 3:**
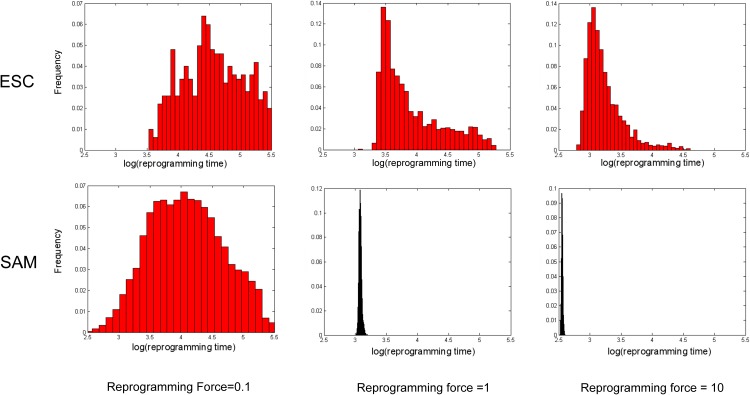
Reprogramming time distributions for various Oct4 and WUS over-expression levels. Comparison of the time it takes to reprogram a cell in the ESC model (first row) and the SAM model (second row). The three columns represent over-expression 0.1, 1 and 10 respectively. We conducted independent simulations for each over-expression level and plotted the distributions of monitored reprogramming times.

We monitored the reprogramming time for the ESC (Oct4-Nanog incoherence is present) and SAM model (Fig 3). The reprogramming time distributions show that, at least for high over-expression, the SAM model reprograms faster compared to the ESC model. The reprogramming time distributions obtained from ESC model simulations are more skewed than the ones obtained from SAM model, due to the presence of incoherent loop between Oct4 and Nanog. Also, the variations in reprogramming time are larger for the ESC model.

Altogether, our results suggest that the gene regulatory networks topologies along with the differentiation dynamics are similar for the SAM and ESC systems. However, the reprogramming dynamics differ, the SAM model being more amenable to reprogram from a differentiated state.

## Discussion

It appears that simplified single cell models of the mammalian embryonic stem cells and plant stem cells can account for the differences in reprogramming properties, although the network dynamics are similar when it comes to differentiation properties. The ‘simple’ reactivation of CLV3/WUS is in nice agreement with plants’ capability to spontaneously create new stem cells in the initialization of new reproductive organs around the main stem [4], as discussed above. This indicates that many of the interactions taking part in the cell state transitions could be simplified to the few components used here in the core regulatory modules. It should be stressed that the Oct-dependent epigenetic effect is included in the ESC model [38]. In contrast such epigenetic mechanism has still not been identified when overexpressing WUS in the SAM [18]. In both kingdoms, over-expression of key genes can be used as a reprogramming recipe [24, 42]. One could also accomplish similar results by suppressing the differentiation gene G. On the other hand since there is very likely more than one differentiation gene in the systems, such an approach might not be sufficient alone, as can be exemplified by the relatively weak phenotypes of the KAN loss of function mutations. Combining over-expression and suppression should lead to a more efficient reprogramming. Indeed, recent experimental results showed that depleting Mbd3, a core member of the Mbd3/NuRD (nucleosome remodelling and deacetylation) repressor complex, together with transcription factors over-expression dramatically improve reprogramming efficiency [47]. Mbd3/NuRD plays also a key role in reprogramming and its increased levels can enhance reprogramming efficiency when co-expressed with reprogramming factor NANOG [48]. The small variations of wiring in the core networks of the two stem cell kingdoms could be due to players, which appear to be specialized, being actually able to hold multiple roles in the mammalian stem cell system e.g. Oct4 [40, 49], Mbd3 [47, 48], while plants are known to use different proteins from the same protein families in regulating differentiation in different tissues [25]. In particular, we found that the incoherent feed forward motif between Oct4 and Nanog could be perturbed, leading to more similar reprogramming behaviour as in plants. For example, a reduction of the repression would lead to more plant like behaviour. In plants, this repression sets the WT regulation of number of stem cells and if this is reduced, spontaneous differentiation appears.

In conclusion, by only studying the dynamics of core modules for stem cell regulation within these simplified SAM and ESC models a clear message emerges—the small variations in wiring in the core regulatory networks can explain why reprogramming is more natural in the plant than the mammalian world. For plants reprogramming is a much-needed competence in contrast to animals where it should be more prohibited.

## Methods

### The shoot apical meristem model

The plant SAM gene circuit in Fig 1A is based on following interactions: CLV represses WUS [32, 33], WUS positively regulates CLV [5], G_SAM_ (KAN1) and WUS are repressing each other [18].

The corresponding set of equations are obtained for the dynamics of CLV, WUS and G_SAM_ (KAN) with mRNA and protein concentrations denoted by [W], [C], [GSAM] and [w], [c], [g_SAM_] respectively.

$$\frac{d\left[W\right]}{dt}=\frac{p_{0}}{1+p_{1}\left[C\right]+p_{2}{\left[g_{SAM}\right]}^{2}}-d_{W}\left[W\right]$$

$$\frac{d\left[w\right]}{dt}=P_{w}\left[W\right]-d_{w}\left[w\right]$$

$$\frac{d\left[C\right]}{dt}=\frac{p_{3}+p_{4}\left[w\right]}{1+p_{4}\left[w\right]}-d_{C}\left[C\right]$$

$$\frac{d\left[c\right]}{dt}=P_{c}\left[C\right]-d_{c}\left[c\right]$$

$$\frac{d\left[G_{SAM}\right]}{dt}=\frac{p_{5}}{1+p_{6}{\left[w\right]}^{2}}-d_{G_{SAM}}\left[G_{SAM}\right]$$

$$\frac{d\left[g_{SAM}\right]}{dt}=P_{g_{SAM}}\left[G_{SAM}\right]-d_{g_{SAM}}\left[g_{SAM}\right]$$

We use mass action dynamics for protein production and degradation, and a standard Shea-Ackers description for the transcriptional regulation [50], which adds specific constraints on parameters for the underlying elementary reactions for exact description of dynamics and noise distributions [51]. *P*_*x*_ represents production rates of protein *x*, *d*_*x*_ is the degradation rate of molecule *x* (mRNA and protein), and the *p* parameters relate to the transcriptional regulation. Three examples of parameters sets from our optimizations (see Parameter optimization section) that were used for calculating the reprogramming efficiencies depicted in Fig 2 are shown below in Table 1. It must be noted that we optimized the parameters based on two main constraints: bistability and spontaneous differentiation. For the SAM model we found 43 successful parameter sets, see Table A in S1 File. The protein production rates P and the mRNA and protein decay rates d were chosen to be 0.01.

**Table 1:** Three SAM parameters sets examples out of 43 parameter sets that were optimized based on bistability and spontaneous differentiation constraints.

Parameters	p_0_	p_1_	p_2_	p_3_	p_4_	p_5_	p_6_
Set 1	0.11	0.05	5.58	0.04	0.08	0.84	0.002
Set 2	0.04	0.02	3.63	0.003	1.90	1.03	0.02
Set 3	0.05	0.74	4.57	0.03	0.49	0.78	0.01

### The embryonic stem cell model

The mammalian embryonic stem cell circuit in Fig 1A was built based on following: OCT4 and SOX2 form a complex which induces its own production [34] and also induces and represses NANOG and induces G_ESC_ [2, 3, 38], NANOG represses itself [35, 36], G_ESC_ (Gata6, Sox17) and NANOG are mutually repressive furthermore G_ESC_ represses OCT4/SOX2 [38]. We put forward the following set of ordinary differential equations from a thermodynamic approach [50] describing the behaviour of NANOG, OCT4/SOX2 and the differentiation gene G_ESC_ (GATA6; SOX17) with mRNA and protein concentrations denoted by [N], [OS], [G_ESC_] and [n], [os], [g_ESC_] respectively.

$$\frac{d\left[N\right]}{dt}=\frac{p_{1}\left[os\right]\left(p_{0}+p_{1}\left[os\right]\right)}{1+p_{1}\left[os\right]\left(p_{1}\left[os\right]+p_{2}\left[n\right]+p_{3}\left[os\right]\right)+p_{4}\left[os\right]{\left[g_{ESC}\right]}^{2}}-d_{N}\left[N\right]$$

$$\frac{d\left[n\right]}{dt}=P_{n}\left[N\right]-d_{n}\left[n\right]$$

$$\frac{d\left[OS\right]}{dt}=\frac{p_{5}+p_{6}{\left[os\right]}^{2}}{1+p_{6}{\left[os\right]}^{2}+p_{7}{\left[g_{ESC}\right]}^{2}}-d_{OS}\left[OS\right]$$

$$\frac{d\left[os\right]}{dt}=P_{os}\left[OS\right]-d_{os}\left[os\right]$$

$$\frac{d\left[G_{ESC}\right]}{dt}=\frac{p_{8}+p_{9}{\left[g_{ESC}\right]}^{2}+p_{10}\left[os\right]}{1+p_{9}{\left[g_{ESC}\right]}^{2}+p_{10}\left[os\right]+p_{11}{\left[n\right]}^{2}}-d_{G_{ESC}}\left[G_{ESC}\right]$$

$$\frac{d\left[g_{ESC}\right]}{dt}=P_{g_{ESC}}\left[G_{ESC}\right]-d_{g_{ESC}}\left[g_{ESC}\right]$$

Parameters are defined as for the SAM model. Three examples of parameters sets from our optimizations (see Parameter optimization section below) used for the calculations of reprogramming efficiencies depicted in Fig 2 are shown in Table 2. It should be mentioned that these sample parameter sets were extracted from wider sets indicating that the solutions indeed are not local. For the ESC model 25 parameter sets accounted for bistability and spontaneous differentiation in the system see Table B in S1 File. The protein production rates P and the mRNA and protein decay rates d were considered to be 0.01 for all species.

**Table 2:** Three ESC parameters sets examples out of 25 parameter sets that were optimized based on bistability and spontaneous differentiation constraints.

Parameters	p_0_	p_1_	p_2_	p_3_	p_4_	p_5_	p_6_	p_7_	p_8_	p_9_	p_10_	p_11_
Set 1	50	1.50	0.40	0.90	0.40	0.01	1.00	1.00	0.005	0.05	1.20	0.35
Set 2	50	1.60	0.60	0.90	0.32	0.007	1.34	1.00	0.005	0.04	1.55	0.35
Set 3	50	1.71	0.42	0.90	0.30	0.006	0.38	0.30	0.005	0.08	1.20	0.35

### Differentiation simulations

For both SAM and ESC gene regulatory networks we developed computational models consisting of ordinary differential equations shown in the sections above. The Shea- Ackers approach [50] was used for describing transcription while mass action kinetics were employed for describing mRNA and protein degradation along with production of protein. We consider the initial conditions of the system to be the state where all the genes have high expression except G_SAM_ and G_ESC_ (stem cell state). To get familiar with the models and their normal behaviour several stochastic simulations were conducted [52], two examples corresponding to two single cells are presented in Fig 1B. We also conducted multiple simulations corresponding to cells colonies. Fig A in S1 File shows WUS and OCT4-SOX2 gene expression distribution at a time point towards the end of the simulations. The distributions demonstrate the robust bistability of the SAM and ESC systems. The parameters used for producing the results shown in Fig 1B and Fig A in S1 File are presented in the first line of Tables 1 and 2. Please note that p_3_ in the ESC model takes a value of 1.5 for these simulations. The spontaneous differentiation time for both models was investigated. The differentiation time was taken to be time interval between start of simulation and the moment that both the types of mRNA, characteristic for the stem cell state (e.g. OCT4/SOX2 and NANOG for ESC), had lower levels than the differentiation promoting genes. Before saving the differentiation time, the simulation ran for yet 10^4^ reactions to separate short fluctuations from real state shifts. If the system remained in the differentiated state for these 10^4^ reactions we consider the simulation to be a successful simulation of the differentiation process.

We conducted 100 stochastic simulations for each parameter set, fixing a maximum number of reactions equal to 10^7^, as a stopping criterion. Matlab (The Mathworks) was used to solve the differential equations and conduct stochastic Gillespie simulations.

### Reprogramming simulations

We initiate our simulations by considering the systems to be in a state where G_SAM_ and G_ESC_ are at high values (differentiated state) while the rest of the genes (the stem cell specific ones) are at low values. The promotion of stem cell specific genes was implemented by adding a constant production (reprogramming force) to the equation for OCT4/SOX2 mRNA rate of change in the ESC model and correspondingly for WUS in the SAM model. If the addition of this constant resulted in a switch of the system to a state where G_SAM_ and G_ESC_ expression are low while the other genes expression are high then we considered the reprogramming to be successful. The reprogramming force of over-expression level for OCT4/SOX2 and WUS were varied in a logarithmic fashion. For each value of over-expression level, simulation data was collected to measure the degree of success (the efficiency) for the reprogramming method i.e. the time required to reprogram the cell and the fraction of reprogrammed cells. Initially, 100 simulations were performed for each parameter set and each value of reprogramming force with the stop criterion of 10^6^ reactions. In order to gather statistics 400 additional simulations were made for the reprogramming force shown in Fig 2.

### Parameter optimization

To optimize the parameter sets for the SAM and ESC models we used the simulated annealing global optimization algorithm [53, 54]. We optimized the parameters based on the constraints that the systems must be bistable and spontaneous transition from pluripotent state to differentiated state must occur during stochastic simulations. To take into account the bistability of the models two cost functions were needed, one for the stem cell state and another one for the differentiated state. The stem cell state is defined as in [38] as a state with high levels of WUS, CLV3, OCT4/SOX2, NANOG and low levels of G_SAM_ and G_ESC_ and vice versa for the differentiated state. We use the following cost function:

$$f_{stem}=\frac{1}{N}\sqrt{\sum_{i=1}^{N}{\left(x_{stem}^{i,model}-x_{stem}^{i,opt}\right)}^{2}}$$

$$f_{diff}=\frac{1}{N}\sqrt{\sum_{i=1}^{N}{\left(x_{diff}^{i,model}-x_{diff}^{i,opt}\right)}^{2}}$$

$$f_{1}=f_{stem}+f_{diff}$$

where N is the number of different molecules (N = 3), $x_{stem}^{i,model}$ is the mRNA concentration of the species type i (e.g. SAM i = {W, C, G_SAM_}) obtained from the model simulation with initial conditions in the stem cell state, while the $x_{diff}^{i,model}$ is obtained from model simulation starting in differentiated state, $x_{stem}^{i,opt}$ is the optimal value of the concentration of mRNA of species type i.

Although the above cost function specifies the bistability condition a third cost function was introduced for taking into account the differences between stem cell and differentiated cell states:

$$f_{2}=\left|\frac{1}{N}\sqrt{\sum_{i=1}^{N}{\left(x_{stem}^{i,opt}-x_{diff}^{i,opt}\right)}^{2}}-\frac{1}{N}\sqrt{\sum_{i=1}^{N}{\left(x_{stem}^{i,model}-x_{diff}^{i,model}\right)}^{2}}\right|$$

The second cost function (f_2_) penalized solutions where only one of the states was fitted well. Furthermore it facilitated to distinguish between successful and unsuccessful optimizations. x^i,model^ was obtained from conducting model simulations using a fourth order Runge-Kutta with adaptive step size method. x^i,opt^ values resemble experimental observations with high values varying between 80 and 120 and the low value between 0 and 20.

We used an annealing schedule similar to the one in [54]. We define the acceptance ratio as:

$$X\left(c\right)=\frac{number\;of\;accepted\;transitions}{number\;of\;proposed\;transitions}$$

where c is the control parameter c_new_ = k_cool_c_old_ with k_cool_ = 0.99. The parameter configurations can be viewed as states in a Markov chain. The length of each Markov chain should be large enough for the algorithm to be able to explore the cost function landscape around the point defined by the configuration obtained in the previous step in the cooling process. The higher dimension a problem has, the longer the chain should be. This feature is incorporated in the following expression for the length: L = L_0_n, with standard length L_0_ = 10 and n being the dimension of the problem.

## Supporting information

S1 File Contains Table A with all optimized parameters for the SAM model, Table B with all optimized parameters for ESC model, Table C showing sensitivity analysis results and Fig A showing distribution results from population level stochastic simulations. (PDF)
