# Supplementary material for: Different reprogramming propensities in plants and mammals: Are small variations in the core network wirings responsible?
Source: PLoS One. 2017 Apr 6;12(4):e0175251. doi: 10.1371/journal.pone.0175251 (PMC5383272; doi:10.1371/journal.pone.0175251)

Different reprogramming propensities in plants and mammals:
Are small variations in the core network wirings responsible?

Supplementary Information

Victor Olariu[1, 2], Julia Nilsson[1], Henrik Jönsson[1, 3*], Carsten Peterson[1*]

1. Computational Biology and Biological Physics, Lund University, Lund, Sweden,

2. Center for Models of Life, Niels Bohr Institute, University of Copenhagen, Copenhagen, Denmark

3. Sainsbury Laboratory, University of Cambridge, Cambridge, United Kingdom

Table A. The SAM system parameter sets that were successfully optimized based on bistability and spontaneous differentiation constraints.

Parameters	p_0	p_1	p_2	p_3	p_4	p_5	p_6
set1	5.584	4.253e-02	8.229e-02	1.159e-01	5.427e-02	8.454e-01	2.594e-03
set2	5.471	3.762e-02	2.338e-02	7.239e-07	7.025e-02	8.675e-01	1.101e-03
set3	3.634	2.744e-02	1.904	4.282e-02	2.277e-02	1.031	2.075e-02
set4	4.887	3.157e-02	5.03e-02	2.994e-02	2.017e-01	1.14	4.446e-03
set5	6.067	4.326e-02	1.905e-01	1.061e-01	2.035e-01	1.124	1.251e-02
set6	4.183e+01	3.599e-01	8.863e+01	1.538e-01	1.9e-01	1.126	9.202e-02
set7	3.549	3.435e-02	4.138e-01	1.77e-03	3.646e-02	9.911e-01	1.066e-02
set8	4.572	3.487e-02	4.965e-01	5.598e-02	7.44e-01	7.829e-01	1.59e-02
set9	5.369	4.289e-02	6.224e-01	4.655e-02	8.617e-01	7.803e-01	1.744e-02
set10	3.057	3.419e-02	1.847e-01	1.652e-01	4.288e-01	1.191	2.02e-02
set11	7.756	9.357e-02	2.492e+01	1.321e-01	4.015e-02	9.481e-01	5.823e-02
set12	3.972	2.938e-02	2.195e-01	8.167e-02	1.015e-01	8.995e-01	7.124e-03
set13	4.999	3.996e-02	4.731e-01	8.776e-02	1.09e-01	8.701e-01	1.13e-02
set14	4.246	2.751e-02	1.045e-01	1.84e-01	4.299e-02	9.852e-01	3.91e-03
set15	2.778	1.32e-02	4.504e-03	2.402e-03	5.006e-02	1.062	1.047e-03
set16	8.714	6.36e-02	2.361e+01	1.917e-01	3.884e-02	9.73e-01	3.046e-02

Parameters	p_0	p_1	p_2	p_3	p_4	p_5	p_6
set17	6.479	6.945e-02	6.059e-02	8.135e-02	1.971e-01	7.786e-01	2.939e-03
set18	8.386e+01	9.714e-01	2.102e-01	1.724e-02	8.008e-02	8.666e-01	1.231e-03
set19	2.096	1.618e-02	4.213e-02	2.402e-02	6.818e-01	1.084	1.314e-02
set20	7.507e+01	9.229e-01	1.964	5.329e-02	8.265e-01	1.093	1.105e-02
set21	3.455	1.534e-02	2.112e+01	2.881e-09	2.098e+0	8.986e-01	8.037e-02
set22	9.391	5.66e-02	5.315e+01	9.627e-09	2.412e+0	8.985e-01	5.537e-02
set23	3.896	1.818e-02	2.027e+01	6.99e-09	2.029e+0	9.035e-01	6.2e-02
set24	5.984	7.64e-02	7.675e-02	1.214e-02	1.475e-01	1.005	5.773e-03
set25	6.004	6.056e-02	2.037e-01	3.366e-02	6.987e-01	1.096	8.561e-03
set26	5.57	5.906e-02	1.198e-01	1.302e-02	4.424e-01	1.101	1.003e-02
set27	3.5	2.604e-02	1.497e-01	8.518e-02	5.764e-02	1.0	5.538e-03
set28	8.686e+01	8.978e-01	3.762e-01	1.839e-03	6.872e-02	1.041	1.949e-03
set29	1.174e+01	1.271e-01	7.129	3.558e-09	1.858e-02	7.99e-01	1.433e-02
set30	7.253	7.868e-02	2.83e+01	1.227e-08	1.528e-02	9.001e-01	4.292e-02
set31	1.687	1.557e-02	2.036e-01	9.276e-02	2.365e-01	1.092	2.204e-02
set32	7.276e+01	1.144	8.313	9.123e-02	2.349e-01	1.091	3.247e-02
set33	1.362e+01	2.029e-01	1.69	9.336e-02	2.293e-01	1.092	2.733e-02
set34	2.923	3.586e-02	2.168e-01	8.309e-02	1.961e-01	1.102	2.082e-02
set35	1.626	1.512e-02	1.049e+01	1.05e-01	1.801e-01	1.093	1.899e-01
set36	3.268	3.976e-02	1.369e-01	6.35e-02	2.293e-01	1.101	1.207e-02
set37	6.862	9.863e-02	2.17	9.733e-02	1.76e-01	1.101	4.074e-02
set38	1.536	1.387e-02	3.094e-01	9.842e-02	1.976e-01	1.094	4.047e-02
set39	3.046e+01	4.685e-01	2.753	8.62e-02	2.469e-01	1.092	2.436e-02
set40	1.523	1.426e-02	1.764	9.981e-02	1.659e-01	1.101	1.136e-01
set41	2.231	2.569e-02	5.531e-01	9.754e-02	1.863e-01	1.098	5.914e-02
set42	1.301e+01	1.986e-01	5.113e-01	6.569e-02	2.057e-01	1.103	2.025e-02
set43	1.329e+01	1.914e-01	4.854e-01	5.87e-02	2.25e-01	1.101	1.076e-02

Table B. The ESC system parameter sets that were successfully optimized based on bistability and spontaneous differentiation constraints.

Par.	p_0	p_1	p_2	p_3	p_4	p_5	p_6	p_7	p_8	p_9	p_{10}	p_{11}
set 1	5.0e+01	1.5	4.0e-01	0.9	4.05e-01	1.0e-02	1.0	1.0	5.0e-03	5.0e-02	1.2	3.5e-01
set 2	2.50e+01	1.932	3.999e-01	0.9	4.05e-01	1.33e-02	9.51e-01	1.521	1.7e-03	3.12e-02	2.067	4.134e-01
set 3	3.36e+01	2.49	4.0e-01	0.9	4.734e-01	1.18e-02	1.395	1.819	1.5e-02	5.0e-02	4.01e-01	1.085e-01
set 4	5.0e+01	1.6	6.05e-01	0.9	3.209e-01	7.5e-03	1.347	1.004	5.8e-03	4.69e-02	1.556	3.5e-01
set 5	3.50e+01	1.692	2.492e-01	0.9	2.362e-01	1.0e-02	1.111	2.995e-01	3.1e-03	5.0e-02	8.0e-01	1.887e-01
set 6	5.0e+01	1.5	4.28e-01	0.9	3.066e-01	6.5e-03	3.85e-01	3.08e-01	5.0e-03	8.85e-02	1.2	3.5e-01
set 7	5.0e+01	1.71	4.0e-01	0.9	4.703e-01	1.0e-02	1.422	7.041e-01	4.6e-03	3.24e-02	1.466	3.511e-01
set 8	5.11e+01	1.784	1.073	0.9	2.534e-01	4.9e-03	1.073	7.306e-01	5.0e-03	3.6e-02	1.661	2.851e-01
set 9	6.58e+01	1.5	5.428e-01	0.9	4.719e-01	6.2e-03	4.49e-01	3.717e-01	7.5e-03	2.63e-02	1.638	3.636e-01
set 10	4.65e+01	1.472	2.221e-01	0.9	5.599e-01	1.0e-02	1.496	6.029e-01	5.6e-03	2.59e-02	1.069	3.403e-01
set 11	4.42e+01	1.544	6.809e-01	0.9	4.05e-01	5.9e-03	1.101	1.0	1.1e-02	2.8e-02	1.081	3.112e-01
set 12	4.09e+01	1.5	4.0e-01	0.9	4.482e-01	1.22e-02	4.64e-01	1.091	3.2e-03	4.02e-02	1.33	3.971e-01
set 13	4.57e+01	1.407	3.64e-01	0.9	3.007e-01	3.1e-03	8.04e-01	4.835e-01	4.2e-03	3.74e-02	1.2	3.5e-01
set 14	5.72e+01	2.183	2.357e-01	0.9	4.05e-01	4.4e-03	1.543	1.186	4.7e-03	4.01e-02	2.048	2.614e-01
set 15	4.5e+01	0.876	3.789e-01	0.9	1.255e-01	1.66e-02	1.0	1.922	3.9e-03	3.1e-02	1.147	3.392e-01
set 16	5.62e+01	1.527	5.071e-01	0.9	3.839e-01	1.49e-02	1.234	8.072e-01	3.3e-03	3.65e-02	1.2	2.854e-01
set 17	5.0e+01	1.236	3.205e-01	0.9	2.68e-01	9.0e-03	1.119	6.705e-01	2.6e-03	6.2e-02	6.60e-01	2.692e-01
set 18	6.74e+01	1.122	4.933e-01	0.9	4.323e-01	1.26e-02	1.099	1.572	2.0e-03	2.04e-02	1.104	3.5e-01
set 19	4.17e+01	1.095	3.106e-01	0.9	2.68e-01	1.25e-02	5.41e-01	1.0	5.9e-03	7.06e-02	7.08e-01	3.538e-01
set 20	4.80e+01	1.12	3.779e-01	0.9	3.529e-01	8.6e-03	1.058	4.914e-01	3.6e-03	3.75e-02	6.50e-01	2.462e-01
set 21	1.81e+01	1.493	3.144e-01	0.9	3.668e-01	7.9e-03	6.39e-01	1.0	2.3e-03	3.76e-02	1.054	4.423e-01
set 22	2.73e+01	1.5	3.165e-01	0.9	4.05e-01	9.9e-03	1.92e-01	1.003	4.7e-03	5.47e-02	1.2	4.747e-01
set 23	5.39e+01	1.5	5.147e-01	0.9	2.192e-01	1.19e-02	1.176	9.546e-01	6.1e-03	2.22e-02	2.349	3.5e-01
set 24	2.55e+01	1.909	2.353e-01	0.9	4.529e-01	6.2e-03	7.37e-01	1.031	2.5e-03	6.54e-02	1.2	3.5e-01
set 25	4.01e+01	2.17	4.0e-01	0.9	3.833e-01	5.1e-03	1.381	4.783e-01	5.0e-03	6.57e-02	1.484	2.866e-01

Table C. The sensitivity analysis results for the ESC system parameters. The dark blue rows show the parameters that influence the most the reprogramming efficiency.

	**Reprogramming Efficiency**		
Parameter	*0.5*Parameter*	*Parameter*	*1.5*Parameter*
p_0	0.69	0.71	0.72
p_1	0.15	0.71	0.92
p_2	0.74	0.71	0.69
p_3	0.90	0.71	0.27
p_4	0.74	0.71	0.45
p_5	0.70	0.71	0.71
p_6	0.69	0.71	0.73
p_7	0.75	0.71	0.71
p_8	0.73	0.71	0.76
p_9	0.77	0.71	0.49
p_{10}	0.72	0.71	0.73
p_{11}	0.36	0.71	0.75

Fig A. WUS and OCT4-SOX2 gene expressions distributions obtained from multiple single cell simulations.

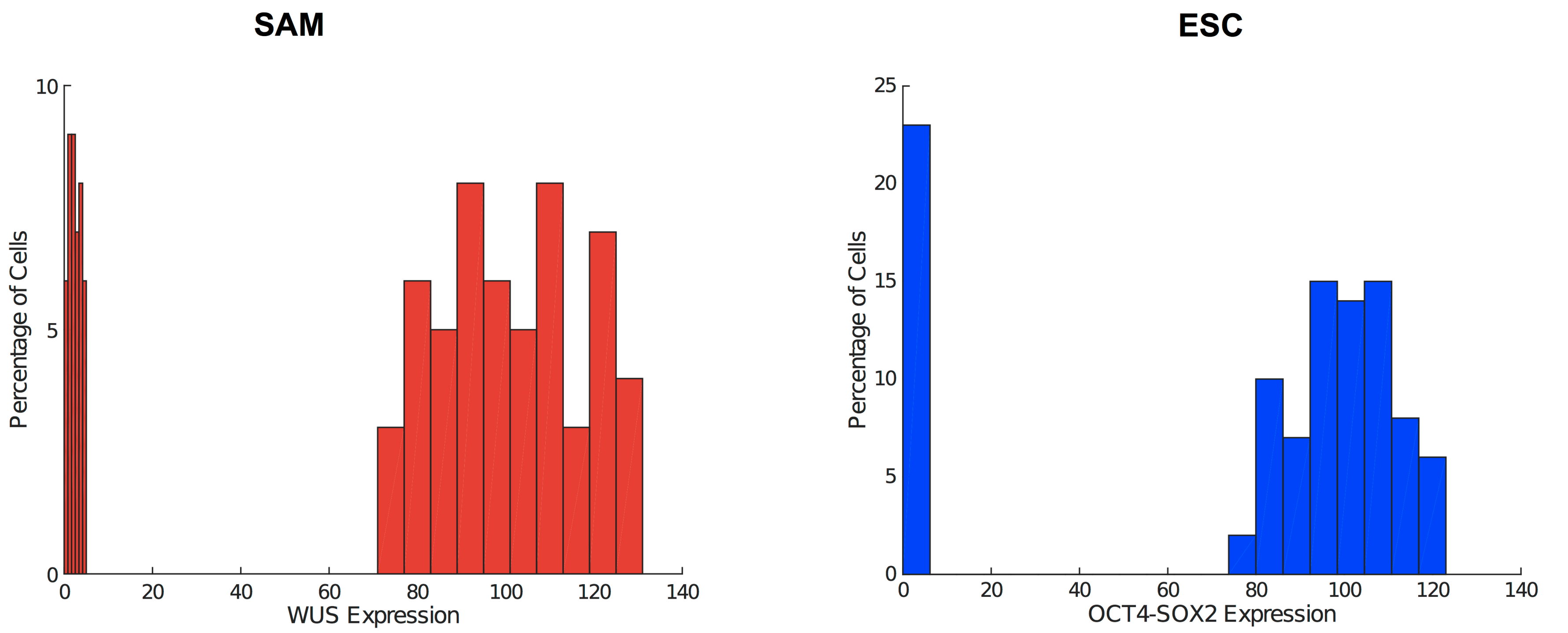

Supplement: S1 File — (PDF) [file pone.0175251.s001.pdf]
